# The Crucial Role of Demannosylating Asparagine-Linked Glycans in ERADicating Misfolded Glycoproteins in the Endoplasmic Reticulum

**DOI:** 10.3389/fpls.2020.625033

**Published:** 2021-01-12

**Authors:** Jianjun Zhang, Jiarui Wu, Linchuan Liu, Jianming Li

**Affiliations:** ^1^State Key Laboratory for Conservation and Utilization of Subtropical Agro-Bioresources, College of Forestry and Landscape Architecture, South China Agricultural University, Guangzhou, China; ^2^Guangdong Key Laboratory for Innovative Development and Utilization of Forest Plant Germplasm, College of Forestry and Landscape Architecture, South China Agricultural University, Guangzhou, China; ^3^Department of Molecular, Cellular, and Developmental Biology, University of Michigan, Ann Arbor, MI, United States

**Keywords:** endoplasmic reticulum, asparagine-linked glycan, endoplasmic reticulum-associated degradation, mannosidase, protein disulfide isomerases

## Abstract

Most membrane and secreted proteins are glycosylated on certain asparagine (N) residues in the endoplasmic reticulum (ER), which is crucial for their correct folding and function. Protein folding is a fundamentally inefficient and error-prone process that can be easily interfered by genetic mutations, stochastic cellular events, and environmental stresses. Because misfolded proteins not only lead to functional deficiency but also produce gain-of-function cellular toxicity, eukaryotic organisms have evolved highly conserved ER-mediated protein quality control (ERQC) mechanisms to monitor protein folding, retain and repair incompletely folded or misfolded proteins, or remove terminally misfolded proteins via a unique ER-associated degradation (ERAD) mechanism. A crucial event that terminates futile refolding attempts of a misfolded glycoprotein and diverts it into the ERAD pathway is executed by removal of certain terminal α1,2-mannose (Man) residues of their *N*-glycans. Earlier studies were centered around an ER-type α1,2-mannosidase that specifically cleaves the terminal α1,2Man residue from the B-branch of the three-branched N-linked Man_9_GlcNAc_2_ (GlcNAc for *N*-acetylglucosamine) glycan, but recent investigations revealed that the signal that marks a terminally misfolded glycoprotein for ERAD is an *N*-glycan with an exposed α1,6Man residue generated by members of a unique folding-sensitive α1,2-mannosidase family known as ER-degradation enhancing α-mannosidase-like proteins (EDEMs). This review provides a historical recount of major discoveries that led to our current understanding on the role of demannosylating *N*-glycans in sentencing irreparable misfolded glycoproteins into ERAD. It also discusses conserved and distinct features of the demannosylation processes of the ERAD systems of yeast, mammals, and plants.

## Introduction

**FIGURE 1:**
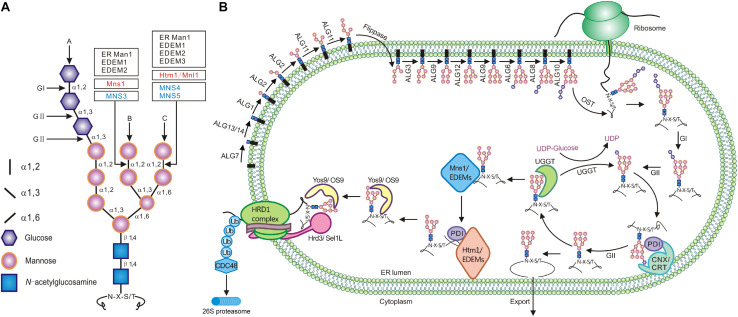
The synthesis of *N*-glycan and its role in ERQC. **(A)** The structure of the three-branched Glc_3_Man_9_GlcNAc_2_. The inlet lists signs/shapes for different sugars and glycosidic linkages. Vertical arrows mark three different branches while the horizontal arrows indicate the cleavage sites of glucosidases and mannosidases. **(B)** The assembly of the *N*-glycan precursor starts at the cytosolic side of the ER membrane with addition of two GlcNAc residues to the membrane-anchored Dol-P linker followed by sequential attachment of the β1,4Man residue (catalyzed by ALG1), the α1,3Man/α1,6Man residues (by ALG2), and the two α1,2Man residues (by ALG11). The resulting Man_5_GlcNAc_2_-PP-Dol is flipped into the ER lumen where four additional Man residues are sequentially added from Dol-P-Man donor (catalyzed by ALG3, ALG9, ALG12, and ALG9) to generate Man_9_GlcNAc_2_-PP-Dol. ALG6, ALG8, and ALG10 catalyze the sequential addition of three Glc residues to form Glc_3_Man_9_GlcNAc_2_ that is *en bloc* transferred by OST onto certain Asn residues of nascent polypeptides. Immediately after the transfer, GI and GII rapidly remove the terminal and middle Glc residues to generate GlcMan_9_GlcNAc_2_. This *N*-glycan is recognized and bound by CNX/CRT that recruit additional chaperones and folding catalysts to assist the folding of those monoglucosylated glycoproteins. The removal of the last Glc residue by GII releases the glycoproteins from CNX/CRT. A correctly folded glycoprotein is demannosylated on the B-branch by Mns1/ERManI while trafficking to the Golgi apparatus whereas a misfolded/incompletely folded glycoprotein is recognized/bound by UGGT that adds back a Glc residue to regenerate GlcMan_9_GlcNAc_2_, forcing its reassociation with CNX/CRT for refolding. If a misfolded glycoprotein stays in the ER too long for engaging multiple futile refolding attempts, its *N*-glycans are slowly demannosylated by Mns1/ERManI and members of the Htm1/EDEM family (likely forming a disulfide bridged complex with members of the PDI family), generating *N*-glycans with an exposed α1,6Man residue. The ERAD lectin (OS-9/Yos9/EBS6) recognizes/binds α1,6Man-exposed *N*-glycans and works together with Hrd3/Sel1L/EBS5, which binds surface-exposed hydrophobic residues, to bring an irreparable misfolded glycoprotein onto the ER membrane-anchored ERAD complex containing a ubiquitin ligase (such as Hrd1) and its accessary factors. This complex not only ubiquitinates but also retrotranslocates a committed ERAD client that is subsequently escorted into the cytosolic proteasome for its complete degradation.

Secretory and transmembrane proteins of eukaryotic organisms are synthesized on cytosolic ribosomes and enter the endoplasmic reticulum (ER) for their folding and maturation (Rapoport, 2007). Most of those nascent polypeptides are co-/post-translationally modified by asparagine-linked glycosylation (*N*-glycosylation) with three-branched Glc_3_Man_9_GlcNAc_2_ (Glc, Man, and GlcNAc denoting glucose, mannose, and *N*-acetylglucosamine, respectively) (Figure 1A). It has been well established that *N*-glycosylation is critical for attaining correct protein conformations by increasing thermodynamic stability, marking segments for surface exposure, and recruiting various lectins and their associated chaperones and folding catalysts (Helenius and Aebi, 2004; Wang et al., 2015). However, protein folding (especially for multi-spanning membrane proteins) is an inefficient and error-prone process that is constantly affected by genetic mutations, transcriptional and translational errors, stochastic cellular events, and a wide range of environmental stresses, resulting in accumulation of misfolded proteins in the ER. Misfolding a protein not only reduces its own activity but often exerts a dominant negative impact on its interacting proteins to augment its deleterious effect on cell physiology. Fortunately, eukaryotic organisms are equipped with a wide range of protein quality control mechanisms that recognize various folding defects, repair and refold misfolded proteins, and degrade irreparable misfolded proteins to maintain protein homeostasis of various cellular compartments (Ellgaard et al., 1999; Balchin et al., 2016).

One of the best studied protein quality control mechanisms is the ER protein quality control (ERQC) system that relies on protein *N*-glycosylation to monitor protein folding status, retain incompletely folded and misfolded glycoproteins in the ER, repair folding defects, and/or retrotranslocate irreparable misfolded proteins into the cytosol for proteasome-mediated proteolysis (Xu and Ng, 2015). This unique degradation process is widely known as ER-associated degradation (ERAD) that is highly conserved in eukaryotic organisms from yeast to plants and human (Stevenson et al., 2016; Berner et al., 2018; Strasser, 2018). The pathway consists of 4 interdependent steps: client recognition and recruitment, ubiquitination, retrotranslocation, and delivery to the cytosolic proteosome for proteolysis. Recent studies have shown that the ERAD machinery builds around several E3 ubiquitin ligases (with each being responsible for a subset of ERAD clients carrying structural defects in their cytosolic, transmembrane, or luminal domains) and contains client recruitment factors and proteins involved in retrotranslocation and substrate delivery to the 26S proteasome.

Given the high energy costs of protein synthesis (Buttgereit and Brand, 1995), eukaryotic cells prioritize repair/refolding over protein degradation. However, continuous futile refolding attempts would titrate away chaperones and folding catalysts that are needed to fold newly synthesized proteins that continuously enter into the ER, potentially reducing the ER folding capacity, increasing ER accumulation of misfolded and incompletely folded proteins, and disrupting ER proteostasis. Thus, eukaryotic cells have to make a crucial life-or-death decision to terminate futile folding attempts and to deliver those irreparable misfolded glycoproteins into the ERAD pathway. It is believed that a terminally misfolded protein is extracted from its futile folding cycles via slow actions of several highly conserved α1,2-mannosidases, which remove key terminal α1,2Man residues of *N*-glycans on the misfolded protein with a prolonged ER residence. Such a theory was widely known as the “mannose/mannosidase timer” hypothesis that was initially proposed in 1994 (Helenius, 1994). It was previously thought that removing the terminal α1,2Man residue of the B-branch of Man_9_GlcNAc_2_ glycans by the ER-type α1,2-mannosidase marks a terminally misfolded protein for degradation. However, further studies have shown that although the α1,2Man-trimming of the B-branch of *N*-glycans on misfolded glycoproteins is a necessary step, the actual *N*-glycan ERAD signal is created by the cleavage of the α1,2Man-α1,6Man linkage of the C-branch to expose the hidden α1,6Man residue that can be subsequently recognized and bound by a highly conserved ERAD lectin, known as OS-9 for osteosarcoma amplified 9 in mammalian cells and Yos9 (yeast homolog of OS-9) in yeast (Figure 1). OS-9/Yos9 works together with Sel1L/Hrd3 (Suppressor of lin-12-Like 1 in mammals and its homolog HMG-CoA reductase degradation 3 in yeast, respectively), which recognizes surface-exposed hydrophobic residues, to take the misfolded glycoprotein to the ER membrane-anchored ERAD complex for its ubiquitination, retrotranslocation, and cytosolic delivery to the 26S proteosome for complete proteolysis (Figure 1). In the past 27 years since the initial revelation of a potential role of Man-trimming in glycoprotein degradation (Su et al., 1993), many studies were directed toward understanding the biochemical functions of the Man-trimming reactions and identifying mannosidases that cleave terminal α1,2Man residues in a branch-specific manner. Because most ERAD studies, especially those on the role of *N*-glycan demannosylation in ERAD, were performed in yeast (*Saccharomyces cerevisiae*) and cultured mammalian cells, we attempt to provide plant biologists a historical review on the major discoveries that have progressively enhanced our understanding of a key event of the ERQC mechanism to tip the balance of repair/refolding-removal/degradation of misfolded glycoproteins. Our review also discusses the research progress of the plant ERAD study in comparison with experiments performed in the yeast and mammalian cell cultures. Before we survey the relevant literature on the Man-trimming reactions, we first briefly discuss *N*-glycosylation and one unique glycoprotein folding process known as the calnexin/calreticulin (CNX/CRT) cycle.

## *N*-Glycosylation in the ER

The *N*-glycosylation is one of the most common protein post-translational modifications, and more than 70% of secretory/transmembrane proteins in human cells are *N*-glycosylated after they enter into the ER. *N*-glycosylation is initiated by a single-step *en bloc* transfer of a preassembled Glc_3_Man_9_GlcNAc_2_ from its lipid carrier dolichyl pyrophosphate (Dol-PP) to select asparagine (Asn or N) residues within the Asn-X-Ser/Thr sequon (X indicating any amino acid except proline while Ser/Thr denoting serine/threonine residue) of a nascent polypeptide. This transfer reaction is catalyzed by a multisubunit enzyme complex known as oligosaccharide transferase (OST) (Strasser, 2016; Harada et al., 2019) (Figure 1). The assembly of the three-branched Dol-PP-Glc_3_Man_9_GlcNAc_2_ is a highly conserved pathway involving two topologically distinct sets of glycosyltransfer reactions on both sides of the ER membrane catalyzed sequentially by highly specific glycosyltransferases (Figure 1) (Schachter, 2014; Stanley et al., 2015). On the cytosolic side, an enzyme complex, which consists of Asparagine-Linked Glycosylation 7 (ALG7), ALG13, and ALG14, adds two GlcNAc residues from uridine diphosphate (UDP)-GlcNAc to the membrane-embedded Dol-P linker to make Dol-PP-GlcNAc_2_ (Elbein, 1987; Lu et al., 2012). It is important to note that ALG7 is the target of tunicamycin that is commonly used to induce protein misfolding and ER stress (Heifetz et al., 1979; Yoo et al., 2018). Subsequently, ALG1 (β1,4-mannosyltransferase), ALG2 (a dual-function α1,3/α1,6-mannosyltransferase) and ALG11 (α1,2-mannosyltransferase) sequentially add five mannose residues to generate Dol-PP-Man_5_GlcNAc_2_ (Couto et al., 1984; O’Reilly et al., 2006) (Figure 1). The resulting Dol-PP-glycan is flipped over into the ER lumen, catalyzed by a yet unknown “flippase” that is genetically linked to the yeast *Rtf1* locus (Helenius et al., 2002). The second set of the glycosyltransfer reactions occurs on the luminal side of the ER membrane where 4 additional Man residues are added, catalyzed sequentially by ALG3 (α1,3 mannosyltransferase), ALG9 (α1,2 mannosyltransferase), ALG12 (α1,6 mannosyltransferase), and ALG9 to form Dol-PP-Man_9_GlcNAc_2_ (Aebi et al., 1996; Burda et al., 1996, 1999; Frank and Aebi, 2005) (Figure 1). Three Glc residues are then added to the terminal α1,2-Man residue of the A branch via the three glucosyltransferases (ALG6, ALG8, and ALG10) to generate the final assembly product Dol-PP-Glc_3_Man_9_GlcNAc_2_ (Stagljar et al., 1994; Reiss et al., 1996; Burda and Aebi, 1998; Burda et al., 1999). It should be noted that ALG3/ALG9/ALG12 and ALG6/ALG8/ALG12 use Dol-P-Man and Dol-P-Glc as the sugar donors for their glycosyltransferase reactions, respectively. The assembly process of Glc_3_Man_9_GlcNAc_2_ on the Dol-P linker and its *en bloc* transfer to nascent polypeptides are conserved in mammals and plants (Strasser, 2016).

## The CNX/CRT Cycle for Refolding

Immediately after transferring Glc_3_Man_9_GlcNAc_2_ to an Asn residue of a nascent polypeptide, the terminal and middle Glc residues are removed sequentially by glucosidase I (GI or GCS1) and glucosidase II (GII) (D’Alessio et al., 2010) (Figure 1). The resulting *N*-glycan, GlcMan_9_GlcNAc_2_, is recognized by two ER chaperone-like lectins, a membrane-anchored CNX and its ER luminal homolog CRT (Caramelo and Parodi, 2008). The high-specificity and high-affinity binding between GlcMan_9_GlcNAc_2_ and CNX/CRT is crucial for folding a nascent polypeptide as CNX/CRT recruit other ER-chaperones and folding catalysts, including binding immunoglobulin protein (BIP), an ER-localized member of heat shock protein 70 (HSP70) family (Hendershot et al., 1994) and its cochaperones, and protein disulfide isomerases (PDIs) essential for forming inter/intra-molecular disulfide bonds (Kozlov et al., 2010). The chaperone-assisted protein folding is terminated upon removal of the remaining Glc residue by GII, releasing a folded glycoprotein from CNX/CRT (Caramelo and Parodi, 2008). If a glycoprotein folds correctly, it is transported out of the ER to continue its secretory journey. However, if the protein fails to attain its native conformation, it is recognized by UDP glucose:glycoprotein glucosyltransferase (UGGT), an ER-resident protein serving a crucial quality control checkpoint function for thousands of glycoproteins (D’Alessio et al., 2010). UGGT has two functional domains: a large N-terminal domain involved in recognizing misfolded clients via a structurally flexible long arc of 4 thioredoxin-like (TRXL) domains and a smaller highly conserved but structurally rigid C-terminal catalytic domain capable of catalyzing the glucosyltransferase reaction using UDP-Glc as a substrate (D’Alessio et al., 2010; Calles-Garcia et al., 2017; Roversi et al., 2017; Satoh et al., 2017). As a result of the UGGT-catalyzed reglucosylation, the misfolded glycoprotein reassociates with CNX/CRT for another round of chaperone-assisted folding. The alternate reactions of GII and UGGT drive cycles of dissociation and reassociation of a misfolded glycoprotein with CNX/CRT for repeated folding attempts, which is widely known as the CNX/CRT cycle (Hammond et al., 1994), until the glycoprotein acquires its native conformation (Figure 1). However, if the glycoprotein fails to fold correctly within a given time window, it is extracted from the CNX/CRT cycle and diverted into the ERAD pathway for the cytosolic proteasome-mediated proteolysis (Vembar and Brodsky, 2008). Thus, the GII/UGGT-driven CNX/CRT cycle not only helps certain glycoproteins to acquire their native conformations but also provides an ERQC mechanism to recognize, retain/refold, or remove misfolded glycoproteins. Mutation in a mouse UGGT resulted in embryo lethality likely caused by misfolding of some proteins essential for embryogenesis (Molinari et al., 2005). Similarly, mutations of the Arabidopsis UGGT, also known as EBS1 for EMS-mutagenized bri1 suppressor (Jin et al., 2007), result in misfolding and subsequent ERAD of several plant immunity receptors (Li et al., 2009; Saijo et al., 2009; Zhang et al., 2015) but also permit the plasma membrane-localization of bri1-9, an ER-retained, misfolded variant of the plant growth receptor Brassinosteroid-Insensitive 1 (BRI1) (Jin et al., 2007). The Arabidopsis studies provide excellent genetic support for a role of the GII/UGGT-driven CNX/CRT cycle in the folding and quality control of glycoproteins (Liu and Li, 2014).

## Importance of Mannose-Trimming in ERAD

What could be the mechanism that terminates the futile folding cycles of an irreparable misfolded glycoprotein to force it into the ERAD pathway? The initial discovery that suggested the importance of *N*-glycan demannosylation in promoting degradation of misfolded glycoproteins came from a mammalian cell culture study showing suppressed degradation of a yeast glycoprotein expressed in cultured mammalian cells by deoxymannojirimycin (dMM) (Su et al., 1993), a known inhibitor of α1,2-mannosidase activity (Elbein, 1987). This mammalian study was confirmed several years later by yeast genetic studies (Knop et al., 1996; Jakob et al., 1998), which demonstrated that deleting the yeast *Mns1* gene, encoding the yeast ER mannosidase I (Camirand et al., 1991), resulted in a reduced degradation of a model yeast ERAD substrate CPY^∗^, an ER-retained mutant variant of the yeast vacuolar carboxypeptidase Y (Finger et al., 1993). Importantly, analyses of degradation rates of CPY^∗^ carrying defined *N*-glycan structures in various yeast mutants (Man_6_GlcNAc_2_ in Δ*alg9*, Man_7_GlcNAc_2_ in Δ*alg12*, and the Man_8_GlcNAc_2_ isoform B lacking the terminal α1,2Man residue of the B-branch in both wild-type and Δ*alg6*, and Man_9_GlcNAc_2_ in Δ*mns1*) suggested that the Mns1-mediated Man-trimming of the B-branch of Man_9_GlcNAc_2_ is important for CPY^∗^ degradation in yeast cells (Jakob et al., 1998). These results led to the “mannose/mannosidase timer” theory (Helenius, 1994; Helenius and Aebi, 2004), hypothesizing that slow action of Mns1 (converting Man9 to Man8B) allows folding intermediates and misfolded glycoproteins to acquire their native conformations without being rushed into the ERAD pathway but removes the B-branch terminal α1,2Man residue of misfolded glycoproteins that stay in the ER for too long engaging hopeless refolding attempts.

Confirmation of the importance of Man-trimming reactions in the mammalian ERAD pathway came years later when many research laboratories (reviewed in Cabral et al., 2001) reported suppressed degradation of a variety of ERAD substrates by dMM and kifunensine (Kif), another widely used inhibitor of α1,2-mannosidases (Elbein et al., 1990), thus extending the “mannosidase timer” hypothesis to the mammalian ERAD mechanism. The major caveat of the model was that it failed to explain how the mammalian ERQC system could differentiate misfolded glycoproteins from their correctly folded conformers that carry the exact same Man8B *N*-glycan known to interact with a well-studied cargo receptor ERGIC53, an ER Golgi intermediate compartment 53-kD protein (Hauri et al., 2000), for their transport into the Golgi apparatus where the remaining α1,2Man residues are sequentially removed by three Golgi-localized α1,2-mannosidases (Moremen et al., 2012). It was subsequently thought that extracting terminally misfolded glycoproteins from the CNX/CRT cycle to force them into the ERAD machinery might involve additional demannosylation steps or require specific ERAD lectin(s) that can recognize both the Man8B glycan and the folding status of a misfolded glycoprotein.

Indeed, further mammalian cell culture studies revealed the presence of Man_5–__7_GlcNAc_2_ *N*-glycans on several mammalian ERAD substrates (reviewed in Lederkremer and Glickman, 2005). More importantly, studies using *N*-glycosylation defective Chinese hamster ovary (CHO) mutant cell lines, in which glycoproteins were *N*-glycosylated with Glc_3_Man_5_GlcNAc_2_ (Villers et al., 1994) or Man_5_GlcNAc_2_ (Ermonval et al., 1997) lacking both the B- and C-branches, demonstrated that ERAD of misfolded glycoproteins could still be suppressed by treatment with α1,2-mannosidase inhibitors (Ermonval et al., 2001; Foulquier et al., 2004). These results suggested that demannosylation beyond the B and C-branch might be required in the mammalian ERAD pathway. It was thought that the Man-trimming of the A-branch prevents the UGGT-catalyzed reglucosylation because the A-branch terminal α1,2Man residue is the Glc-acceptor, thus prohibiting an incompletely folded or misfolded glycoprotein to reenter the CNX/CRT cycle for additional folding cycle and effectively forcing its entry into the ERAD pathway (Lederkremer and Glickman, 2005). Such an explanation was supported by later studies showing that genetic and pharmacological manipulation of the CNX/CRT cycle could alter ERAD of misfolded glycoproteins (Molinari et al., 2003; Oda et al., 2003). It is important to note that extensive demannosylation of *N*-glycans of misfolded glycoproteins was not detected in yeast, which seems to be consistent with the fact that the budding yeast (*Saccharomyces cerevisiae*) lacks UGGT and the CNX/CRT cycle (D’Alessio et al., 2010) and therefore has no need to demannosylate the A-branch of *N*-glycans of irreparable misfolded glycoproteins.

The first indication of a role of Man-trimming in a plant ERAD process was reported in 2001 by a study that investigated degradation of the catalytic A subunit RTA (ricin toxin A subunit) of the ribosome-inactivating cytotoxin ricin when it was expressed in tobacco protoplasts (Di Cola et al., 2001). Ricin is normally produced as a heterodimeric glycoprotein consisting of RTA disulfide bridged with RTB (ricin toxin B subunit) in the seeds of the castor oil plant *Ricinus communis*, and a heterologously expressed RTA without RTB was known to be degraded rapidly via a plant ERAD mechanism (Di Cola et al., 2005). Later studies in Arabidopsis confirmed that exogenous Kif application blocked degradation of at least two ER-retained mutant variants of BRI1, bri1-5 and bri1-9, and a misfolded plant innate immunity receptor, providing additional support for a role of *N*-glycan demannosylation in a plant ERAD process (Hong et al., 2008, 2009; Liebminger et al., 2009; Nekrasov et al., 2009). The Arabidopsis has the GII/UGGT-driven CNX/CRT cycle critical for protein folding and quality control of misfolded glycoproteins (Liu and Li, 2014), but it remains unknown if ERAD of plant glycoproteins involves extensive demannosylation of their *N*-glycans. It is interesting to note that *N*-glycan analysis of an engineered plant ERAD substrate transiently expressed in tobacco leaves revealed the presence of monoglucosylated *N*-glycans with an exposed α1,6Man residue, suggesting that trimming the A-branch terminal α1,2Man residue might be needed to extract a terminally misfolded glycoprotein from the CNX/CRT cycle to force its entry into the ERAD process (Hüttner et al., 2014a). It is also possible that the end-of-life decision of the plant ERQC system might be determined by competition between CNX/CRT and OS9 for binding to glycoproteins carrying *N*-glycans with both refolding and ERAD signals.

## Two Families of Potential ERAD Lectins That Bind the Man-Trimmed *N*-Glycans

The theory of a Man-trimmed *N*-glycan ERAD signal for both yeast and mammalian ERAD pathways prompted intensive searches for ERAD lectins that might recognize the Man8B glycan on misfolded glycoproteins, leading to discovery of two families of proteins (Kanehara et al., 2007). The first one comprises members of the class 1 α-mannosidase family, including yeast Htm1 (homologous to mannosidase 1), mammalian EDEM1-3 (ER degradation-enhancing α-mannosidase-like protein1-3), and Arabidopsis MNS4 and MNS5 (Hüttner et al., 2014b), while the second one is the Yos9/OS-9 family. Both the yeast Htm1 (also known as Mnl1 for mannosidase-like protein) (Hosokawa et al., 2001; Nakatsukasa et al., 2001) and mammalian EDEMs share sequence similarity with yeast Mns1 (α1,2-mannosidase 1) and mammalian ERManI (ER class I α-mannosidase) but lack a cysteine pair (Cys^340^–Cys^385^ in Mns1) that was previously thought to be essential for the yeast Mns1 mannosidase activity (Lipari and Herscovics, 1996). The lack of this conserved cysteine-pair plus all failed initial attempts to demonstrate *in vitro* α1,2-mannosidase activities of Htm1/EDEMs toward free oligosaccharides led to an earlier consensus in the ERAD research field that Htm1/EDEMs were inactive mannosidases that could function as the Man8B-binding lectins (Hosokawa et al., 2001; Jakob et al., 2001; Nakatsukasa et al., 2001; Mast et al., 2005). Consistent with this hypothesis, a Δ*htm1* mutation or RNAi-mediated silencing of EDEM1 inhibited degradation of glycosylated but not non-glycosylated ERAD substrates (Jakob et al., 2001; Nakatsukasa et al., 2001; Molinari et al., 2003), whereas overexpression of Htm1/EDEMs accelerated degradation of glycosylated ERAD clients but had little impact on their non-glycosylated variants (Hosokawa et al., 2001; Molinari et al., 2003; Oda et al., 2003; Mast et al., 2005; Olivari et al., 2005; Hirao et al., 2006). The two EDEM1 studies in 2003 not only demonstrated an interaction of EDEM1 with CNX but also provided strong evidence that EDEM1 enhanced ERAD by extracting misfolded glycoproteins from the CNX/CRT cycle. Overexpression of CNX or inhibition of GII (prolonging the glycoprotein-CNX association) suppressed ERAD, whereas genetic and pharmacological inhibition of the initial creation of GlcMan_9_GlcNAc_2_ *N*-glycan nullified the stimulatory impact of EDEM1 on ERAD of misfolded glycoproteins (Molinari et al., 2003; Oda et al., 2003). Together, these early Htm1/EDEM studies strongly suggested that Htm1/EDEMs could function as ERAD lectins that compete effectively with CNX/CRT to control the repair/refolding-removal/degradation balance of misfolded glycoproteins. It should be noted that although many early studies demonstrated binding of EDEMs with misfolded glycoproteins, no published study had shown that EDEMs interacted with their clients via a glycan-dependent manner except the Hosokawa et al. (2001) study that revealed a slightly stronger EDEM1 binding affinity with its clients carrying the Man8B-glycan than Man9-carrying clients (Hosokawa et al., 2001). In addition to EDEM1, mammalian cells have two other members of the Htm1/EDEM family, EDEM2 and EDEM3, which also stimulated ERAD of glycosylated substrates but not their non-glycosylated variants when overexpressed in cultured mammalian cells (Mast et al., 2005; Olivari et al., 2005; Hirao et al., 2006). Despite strong evidence for the involvement of Htm1/EDEMs in ERAD, the direct experimental support for their suspected lectin function was extremely weak.

The other candidate for an ERAD lectin is Yos9/OS-9 that contain a Man-6-phosphate (Man-6-P) receptor homology (MRH) domain previously implicated in sugar binding. This domain was found to be present in several well studied proteins/enzymes (Munro, 2001), including the γ-subunit of GlcNAc-1-phosphotransferase that generates Man-6-P on lysosomal enzymes whose sorting from the *trans*-Golgi-network to lysosomes is mediated by recognition of the Man-6-P signal (Bao et al., 1996) and the β-subunit of GII involved in removing the 2nd and 3rd Glc residue of the Glc_3_Man_9_GlcNAc_2_ *N*-glycan (Trombetta et al., 1996). The mammalian OS-9 (exhibiting ∼15% sequence identity with Yos9) was initially discovered in 1994 as one of the functionally unknown proteins whose genes were amplified in osteosarcoma (Su et al., 1994) and has a mammalian homolog (with ∼23% sequence identity) known as Erlectin or XTP3-B (XTP3-transactivated gene B) (Cruciat et al., 2006). However, the genetic link of Yos9/OS-9 to ERAD was made 10 years later by a genome-wide screen for yeast deletion mutants defective in ERAD (Buschhorn et al., 2004). This study revealed that a deletion mutation of Yos9 inhibited ERAD of CPY^∗^ but had little impact on the degradation of its non-glycosylated variant. More importantly, it was shown that the Δ*yos9 Δhtm1* double deletion had a more or less similar inhibitory impact on CPY^∗^ ERAD compared to Δ*yos9* or Δ*htm1* single mutations, implying that Yos9 and Htm1 work in the same biochemical pathway for degrading CPY^∗^ in yeast cells. The essential role of Yos9 in the yeast ERAD process was confirmed by three independent studies (Bhamidipati et al., 2005; Kim et al., 2005; Szathmary et al., 2005) and Yos9 was later found to be a component of the yeast ERAD complex containing the ubiquitin ligase Hrd1 (HMG-CoA reductase degradation 1) (Carvalho et al., 2006; Denic et al., 2006; Gauss et al., 2006). The Szathmary et al. (2005) study demonstrated that Yos9 only interacted with CPY^∗^ in wild-type (the predominant *N*-glycan of CPY^∗^ being Man_8_GlcNAc_2_) or Δ*alg3* yeast cells (with ER-localized proteins glycosylated with Man_5_GlcNAc_2_ lacking both B and C branches) but not in yeast cells of Δ*alg9*, Δ*alg12*, and Δ*mns1* (ER-localized proteins glycosylated with Man_6_GlcNAc_2_, Man_7_GlcNAc_2_, and Man_9_GlcNAc_2_, respectively), providing a strong support for Yos9 being an ERAD lectin capable of recognizing and binding Man8B and Man_5_GlcNAc_2_ *N*-glycans. While the Yos9-Man_8_GlcNAc_2_ binding was expected, the Yos9-Man_5_GlcNAc_2_ interaction was really intriguing at the time. More importantly, this study showed that Δ*htm1* significantly reduced the Yos9-CPY^∗^ interaction, confirming that Htm1 works together with Yos9 to recognize a misfolded glycoprotein for ERAD. It is interesting to note that N-glycan analysis of CPY^∗^ of the Szathmary et al. (2005) study revealed the presence of a small percentage of Man_7_GlcNAc_2_-glycan on the CPY^∗^ in wild-type yeast cells. If the researchers had compared the *N*-glycan profiles of CPY^∗^ between wild-type and Δ*htm1* yeast cells, they would have obtained the first genetic evidence for Htm1 being an active α1,2-mannosidase that further demannosylates Man_8_GlcNAc_2_ to generate Man_7_GlcNAc_2_ that can be subsequently recognized by Yos9.

Unlike Yos9 whose protein sequence hints at a localization in the ER, mammalian OS-9 was predicted to have an N-terminal signal peptide without the H/KDEL ER-retrieval motif and was initially thought to be localized on the cytosolic side of the ER membrane, leading to earlier confusions about its biochemical functions (Litovchick et al., 2002; Baek et al., 2005). By contrast, its homolog XTP3-B, which carries an N-terminal signal peptide plus two MRH domains but also lacks the H/KDEL ER retrieval motif, was found to be localized in the ER lumen and was initially implicated in regulating glycoprotein trafficking in an MRH domain-dependent manner, providing the first support of mammalian OS-9/XTP3-B being an ER lectin (Cruciat et al., 2006). A role of OS-9/XTP3-B in ERAD was demonstrated in 2008 when several studies (Bernasconi et al., 2008; Christianson et al., 2008; Hosokawa et al., 2008; Mueller et al., 2008; Alcock and Swanton, 2009) reported that OS-9/XTP3-B is a component of a mammalian membrane-bound ERAD complex that contains HRD1 and Sel1L, the mammalian homologs of yeast Hrd1 and Hrd3, respectively (Lilley and Ploegh, 2005). However, mutational analyses initially suggested that the MRH domain was not directly involved in binding misfolded glycoproteins (Bernasconi et al., 2008; Christianson et al., 2008), which was likely masked by the chaperone activity of OS-9/XTP3-B, but was essential for binding Sel1L (Cormier et al., 2009). However, a later study showed that a mutant OS-9 variant carrying the Arg^188^-Ala lectin mutation in its MRH domain could still bind Sel1L (Hosokawa et al., 2009).

The presence of an OS-9/Yos9 homolog in Arabidopsis was initially reported in 2001 (Munro, 2001), and its corresponding gene was later discovered to be an ER stress-induced gene in a 2003 transcriptomic study (Martinez and Chrispeels, 2003). Similar to its mammalian homologs, the Arabidopsis OS9 also lacks the H/KDEL ER retrieval motif and its ER localization likely depends on its interaction with EBS5, the Arabidopsis homolog of the yeast Hrd3/mammalian Sel1L (Liu et al., 2011; Su et al., 2011). Its role as an important ERAD component was confirmed through forward and reverse genetic approaches (Hüttner et al., 2012; Su et al., 2012). Loss-of-function mutations in AtOS9, which is the *Arabidopsis thaliana* homolog of OS-9 (Hüttner et al., 2012) and is also known as EBS6 (Su et al., 2012), inhibit ERAD of bri1-5 and bri1-9, leading to their accumulation in the ER and their consequential leakage to the plasma membrane where the two mutant BR receptors can initiate the plant steroid signaling to promote plant growth. Importantly, the interaction of AtOS9/EBS6 with its ERAD clients was shown to be dependent on its MRH domain and Man-trimming of its glycosylated client (Hüttner et al., 2012). A later study revealed that AtOS9/EBS6 could be co-immunoprecipitated with a misfolded mutant variant of the STRUBBELIG (SUB) extracellular domain carrying a Cys^57^-Tyr mutation (SUBEX-C57Y) via a glycan-independent manner (Hüttner et al., 2014a); however, such a glycan-independent AtOS9/EBS6-substrate binding could be mediated by EBS5 known to interact with both ERAD substrates and AtOS9/EBS6. Together, the studies performed in yeast, cultured mammalian cells, and Arabidopsis strongly suggested that Yos9/OS9/AtOS9 are better candidates for the suspected ERAD lectins that binds committed ERAD clients in a MRH/N-glycan dependent manner.

### AN α1,6Man-Exposed *N*-Glycan As The ERAD Signal

Further support for OS-9/Yos9 being a *bona fide* ERAD lectin came from biochemical studies that directly quantified the sugar binding of OS-9/Yos9 by flow cytometry and/or frontal affinity chromatography (FAC). While the flow cytometry analyzes the fluorescent intensity of cultured mammalian cells (displaying different cell surface glycans) stained with fluorescence-decorated OS-9/XTP3-B (directly or indirectly through fluorescently labeled antibodies), the FAC-based assay measures the relative elution volume of a fluorescent-labeled oligosaccharide of defined structures (compared to a control oligosaccharide) from a lectin-immobilized column (Tateno et al., 2007). The application of these two techniques revealed that the recombinant MRH domains of Yos9 or OS-9/XTP3-B (the 2nd MRH domain in XTP3-B) exhibited high affinity binding with high Man-type glycans containing exposed α1,6Man residue but no binding at all with Glc_0–__1_Man_8_GlcNAc_2_ that was previously thought to be the marking signal for ERAD (Quan et al., 2008; Hosokawa et al., 2009; Mikami et al., 2010; Yamaguchi et al., 2010). Importantly, mutating a conserved Arg residue (Arg^188^ in OS-9, Arg^428^ in XTP3-B, and Arg^200^ in Yos9) of the MRH domain greatly diminished the binding of a recombinant MRH domain with the α1,6Man-exposed glycans (Quan et al., 2008; Hosokawa et al., 2009; Mikami et al., 2010; Yamaguchi et al., 2010). These results were consistent with earlier findings that mutations in ALG9 or ALG12 blocked the ERAD of CPY^∗^ in yeast because Δ*alg9* or Δ*alg12* mutation prevents addition of an α1,6Man residue during the assembly of the Dol-PP-Glc_3_Man_9_GlcNAc_2_ (Figure 1) and provided a satisfactory explanation for a previous intriguing finding that Yos9 interacted with CPY^∗^ carrying Man_5_GlcNAc_2_ *N*-glycans in the yeast Δ*alg3* mutant (Szathmary et al., 2005). These *in vitro* MRH-oligosaccharide binding assays prompted *in vivo* testing of the newly discovered ERAD *N*-glycan signal. This was demonstrated beautifully in yeast cells by two genetic approaches: eliminating ALG3 that initiates the ER luminal addition of 4 Man residues and overexpressing ALG12 in a Δ*alg9* mutant. While Δ*alg9* mutation blocked CPY^∗^ degradation, ALG12 overexpression in the Δ*alg9* mutant cells resulted in ∼50% degradation of CPY^∗^, which is consistent with the ratio of Dol-PP-Man_6_GlcNAc_2_ and Dol-PP-Man_7_GlcNAc_2_ produced in the Δ*alg9*/*ALG12-*overexpression strain (Quan et al., 2008). Similarly, a Δ*alg3* mutation, which results in the formation of Man_5_GlcNAc_2_ exposing the first α1,6Man residue attached to the β1,4Man residue (due to lacking both B and C branches), could also stimulate ERAD (Clerc et al., 2009). The revelation of high specificity and high affinity binding of OS-9/XTP3-B with α1,6Man-exposed high Man-type glycans was also consistent with an earlier mammalian cell culture study, which used a mutant CHO cell line whose proteins were glycosylated with either Man_5_GlcNAc_2_ or Man_9_GlcNAc_2_ to conclude preferential degradation of Man_5_GlcNAc_2_-carrying glycoproteins over Man_9_GlcNAc_2_-bearing glycoproteins (Foulquier et al., 2004). A further support for a crucial role of the α1,6Man-exposed *N*-glycan in the mammalian ERAD pathway came from a recent haploid genetic screening via CRISPR/Cas9 and gene-trap mutagenesis in cultured human KBM7 cells that identified ERAD inhibitory mutations in the human homologs of ALG9 and ALG12 (Timms et al., 2016).

Consistent with the results of the yeast and mammalian studies, loss-of-function mutations in Arabidopsis EBS3 or EBS4 (homologs of the yeast ALG9 and ALG12, respectively) blocked degradation of bri1-5 and bri1-9 (Hong et al., 2009, 2012) and ER-retained mutant variants of two leucine-rich-repeat receptor-like-kinases involved in floral organ abscission (Baer et al., 2016). Importantly, overexpression of EBS4/ALG12 in an *ebs3/alg9* mutant background recreated the Man_7_GlcNAc_2_ *N*-glycan on bri1-5 and bri1-9, thus nullifying the inhibitory impact of the *ebs3/alg9* mutation on the degradation of the two mutant bri1 proteins (Hong et al., 2012). Similar, crossing an Arabidopsis *alg3* mutation, which produced glycoproteins containing Man_5_GlcNAc_2_ exposing a free α1,6Man residue (Henquet et al., 2008; Kajiura et al., 2010), also suppressed the inhibitory impact of *ebs3/alg9* or *ebs4/alg12* mutation on the ERAD of bri1-5 and bri1-9 (Hong et al., 2012). These experiments thus demonstrated that the *N*-glycan signal that tags misfolded proteins for ERAD is conserved between Arabidopsis and yeast/mammalian cells and carries an exposed α1,6Man residue.

## Htm1/EDEMs Are Active Mannosidases *in vivo*

What could be the enzyme(s) responsible for cleaving the C-branch α1, 2Man-α1,6Man linkage to generate the conserved ERAD *N*-glycan signal carrying an exposed α1,6Man residue? Because of their sequence similarity with Mns1/ERManI, a potential role of Htm1/EDEM as active α1,2-mannosidase was investigated right after their initial discoveries in 2001 using free oligosaccharides as substrates with no reported success (Hosokawa et al., 2001; Jakob et al., 2001; Nakatsukasa et al., 2001). However, measuring electromobility changes of glycosylated ERAD substrates on SDS-PAGE (sodium dodecyl sulfate polyacrylamide gel electrophoresis) coupled with *N*-glycan analyses of ERAD clients and total glycoproteins revealed that mammalian EDEMs are active α1,2-mannosidases *in vivo*.

Interestingly, the first member of the Htm1/EDEM family to be demonstrated as an active mannosidase *in vivo* was EDEM3, the last of the three mammalian EDEMs studied (Hirao et al., 2006). EDEM3 is the largest member of the mammalian EDEM family (931 amino acids for the human EDEM3). It has a signal peptide, an α1,2-mannosidase-like domain (MLD), a large C-terminal domain containing a 94-amino acid protease-associated motif, and a C-terminal KDEL ER retrieval motif that is absent in EDEM1 or EDEM2. Consistent with what were previously known for Htm1 and EDEM1/2, EDEM3 not only physically interacted with a widely used mammalian ERAD substrate, NHK that is an ER-retained misfolded α1-antitrypsin variant known as null Hong Kong (Liu et al., 1997), but also promoted NHK degradation. The initial hint for a potential *in vivo* mannosidase activity came from SDS-PAGE analysis of a pulse-chase experiment of ^35^[S]-labeled NHK, revealing a faster-moving NHK band from cultured cells transfected with EDEM3 than that of the non-transfected cells. Importantly, such an EDEM3-induced electromobility shift was eliminated after Kif treatment, suggesting that EDEM3 directly or indirectly demannosylated N-glycans of NHK. Indeed, careful analysis of N-glycans of NHK and total glycoproteins of cultured cells labeled with ^3^[H]Man indicated that overexpression of the wild-type EDEM3 but not its catalytically inactive form EDEM3(E^147^Q) with E^147^ corresponding to E^132^ and E^330^ known to be essential for the α1,2-mannosidase activities of yeast Mns1 and mammalian ERManI, respectively (Vallee et al., 2000a, b), stimulated trimming of *N*-glycans of NHK from Man_8_GlcNAc_2_ to Man_6–__7_GlcNAc_2_ and resulted in a significant increase in Man_6_GlcNAc_2_ with a concomitant decrease in Man_7–8_GlcNAc_2_ of total Endo H-released *N*-glycans (Hirao et al., 2006). The results suggested that EDEM3 is an active mannosidase *in vivo* that is capable of trimming multiple α1,2Mman residues on misfolded and native glycoproteins.

It is interesting to note that earlier EDEM1 studies also observed EDEM1-induced SDS-PAGE mobility changes of glycosylated ERAD substrates during similar pulse-chase experiments (Hosokawa et al., 2001, 2003; Molinari et al., 2003; Mast et al., 2005). However, such mobility changes were thought at the time to be caused by easier access of *N*-glycans of ERAD clients, which were extracted from the CNX/CRT cycle by overexpressed EDEM1, to the B-branch Man-trimming ERManI (Hosokawa et al., 2001; Molinari et al., 2003; Olivari et al., 2005). A similar analysis of Endo H-released *N*-glycans was also performed with the immunoprecipitated NHK from ^3^[H]Man-labeled HEK293 cells transfected with EDEM1 but no increase in Man_6–8_GlcNAC_2_ *N*-glycans was detected (Hosokawa et al., 2003). The failure to detect such increases on NHK in EDEM1-transfected cells could be caused by a combination of EDEM1-enhanced NHK degradation (a much lower amount of NHK to be immunoprecipitated) and a much weaker *in vivo* α1,2-mannosidase activity. Another important result of this study was the detection of a small amount of GlcMan_8_GlcNAc_2_ glycan on the immunoprecipitated NHK from EDEM1-transfected but not non-transfected HEK293 cells; however, its true identity remained unknown till 2010. It should also be important to mention that the first reported EDEM2 study did analyze the total *N*-glycans using ^3^[H]Man-labeled mammalian cells transfected with or without EDEM2 and discovered that the majority of Endo-H-released *N*-glycans were Glc_0–1_Man_9_GlcNAc_2_, leading to a conclusion that EDEM2 was an inactive mannosidase (Mast et al., 2005). The different results in the *N*-glycan analyses of the EDEM2/3 studies could be attributed to distinct characteristics of different cell lines, an inherent limitation of the mammalian cell culture studies. The 2005 EDEM2 study used HEK293 cells derived from human embryonic kidney cells (Mast et al., 2005) while the 2006 EDEM3 experiment used HepG2 cells originated from a human liver cancer tissue (Hirao et al., 2006).

The successful demonstration of EDEM3 exhibiting an *in vivo* mannosidase activity prompted reexamination of EDEM1 that was previously shown to cause similar SDS-PAGE mobility shift of a glycosylated ERAD client (Hosokawa et al., 2001, 2003; Molinari et al., 2003). Careful analysis of the electromobility changes of two ERAD clients, NHK and BACE457 that is an ER-retained splicing-variant of the human β-secretase (Bodendorf et al., 2001; Molinari et al., 2002), revealed that overexpression of the wild-type EDEM1 but not its catalytically dead variant caused Kif-dependent faster mobility of the two ERAD substrates (Olivari et al., 2006), indicating that EDEM1 was also an active mannosidase *in vivo*. Interestingly, EDEM1 also increased the SDS-PAGE mobility of a glycosylated ERAD substrate in mutant CHO cells (B3F7), in which glycoproteins were glycosylated with *N*-glycans lacking both B and C-branches (Cacan et al., 1992), suggesting that EDEM1 was capable of trimming the A-branch α1,2Man residues (Olivari et al., 2006). However, the lack of a negative control (transfecting B3F7 cells with a catalytically dead EDEM1) made it difficult to draw a definitive conclusion on the causative relationship between the observed mobility shift of a misfolded glycoprotein with the suspected A-branch α1,2-mannosidase activity of EDEM1 in B3F7 cells. It is possible that EDEM1 overexpression in B3F7 cells might increase the abundance of other mammalian α1,2-mannosidases capable of cleaving the A-branch terminal α1,2Man residue, such as ERManI and Golgi-localized α1,2-mannosidases that were known to stimulate ERAD when overexpressed in cell cultures (Hosokawa et al., 2003, 2007; Avezov et al., 2008). These results showed that EDEM1 is also an active α1,2-mannosidase *in vivo* that could demannosylate α1,2Man residues of the C/A branches.

Additional support for the suspected α1,2-mannosdase activity of the Htm1/EDEM in promoting ERAD came from two yeast genetic studies. As discussed above, two yeast genetic approaches, one overexpressing ALG12 in a Δ*alg9* mutant and the other deleting ALG3, demonstrated that the true ERAD *N*-glycan signal that marks a misfolded glycoprotein in yeast cells is an *N*-glycan with an exposed α1,6Man residue (Quan et al., 2008). More importantly, both genetic approaches eliminated the requirement of Htm1 for degrading CPY^∗^, implying that Htm1 likely catalyzes the C-branch α1,2Man-trimming reaction to expose the α1,6Man residue. It was a further metabolic study that really confirmed such a speculation (Clerc et al., 2009). Careful analysis of peptide:*N*-glycosidase F (PNGase F)-released ^3^[H]Man-labeled *N*-glycans of total yeast proteins revealed a predominant presence of Man_8_GlcNAc_2_. Importantly, overexpression of Htm1 but not its mutant variants carrying E^222^Q/D^279^N mutations, corresponding to E^214^ and D^275^ essential for the Mns1 activity (Lipari and Herscovics, 1999), resulted in an easily detectable increase in the amount of Man_7_GlcNAc_2_ *N*-glycan, demonstrating that Htm1 was an active α1,2-mannosidase *in vivo*. Consistently, only the wild-type Htm1 plasmid but not its E^222^Q/D^279^N-mutant variants complemented the ERAD-inhibitory Δ*htm1* mutation, confirming that the ERAD-promoting activity of Htm1 absolutely depends on its suspected α1,2-mannosidase activity. This finding was in sharp contrast to what was known about the mammalian EDEM1 whose catalytically inactive mutants were still capable of promoting ERAD (Hosokawa et al., 2010; Ninagawa et al., 2014) likely caused by the demonstrated chaperone function of EDEM1 (Hosokawa et al., 2006; Olivari et al., 2006; Cormier et al., 2009; Kosmaoglou et al., 2009; Termine et al., 2009; Marin et al., 2012; Sokolowska et al., 2015). To determine the branch-specificity of the *in vivo* α1,2-mannosidase activity of Htm1, the Man_7_GlcNAc_2_-glycan produced in Htm1-overexpressing yeast cells was purified and analyzed by *in vitro* digestion with α1,2-exomannosidase that converted Man_7_GlcNAc_2_ to Man_5_GlcNAc_2_. This result, coupled with the ability of overexpressed Htm1 to convert GlcMan_8_GlcNAc_2_-glycan (the A-branch terminal α1,2Man residue being protected by the Glc residue) of the Δ*alg8Δglc2* yeast cells to Glc_1_Man_7_GlcNAc_2_, indicated that Htm1 is a unique α1,2-mannosidase that specifically cleaves the C-branch α1,2Man residue. Thus, the Clerc et al. (2009) study was the first to demonstrate that a member of the Htm1/EDEM family is a C-branch-specific α1,2-mannosidase *in vivo*. This study also revealed that Htm1 was only active toward Glc_0–1_Man_8_GlcNAc_2_ *N*-glycans (lacking the B-branch α1,2Man residue), explaining why the Δ*mns1* mutation completely blocks the ERAD of CPY^∗^ (Knop et al., 1996). The yeast studies not only demonstrated that Htm1 is an active α1,2-mannosidase *in vivo* but also determined its C-branch specificity and its requirement of the Man8B as its substrate.

The C-branch specificity was subsequently confirmed for the mammalian EDEM1 in 2010 when the true identity of the mysterious GlcMan_8_GlcNAc_2_ glycan, which was initially detected by Hosokawa et al. (2003) study that investigated the impacts of overexpressed ERManI or EDEM1 on degradation and Man-trimming of NHK, was determined to be the GlcMan_8_GlcNAc_2_ isomer C (lacking the C-branch terminal α1,2Man residue) (Hosokawa et al., 2010). Importantly, such a unique *N*-glycan was detected on NHK immunoprecipitated from HEK293 cells transfected with the wild-type EDEM1 but not its catalytically inactive mutant, indicating that EDEM1 is a C-branch-specific α1,2-mannosidase capable of directly demannosylating GlcMan_9_GlcNAc_2_ (Hosokawa et al., 2010). A further support for the C-branch specificity of EDEM1 came from analyzing *N*-glycans of total glycoproteins extracted from HepG2 cells transfected with or without EDEM1, showing that EDEM1 overexpression resulted in significant increase in the A isoform of Man_7_GlcNAc_2_ (a terminal α1,2Man residue on the A-branch) and Man_6_GlcNAc_2_ with a concomitant decrease in Man_8_GlcNAc2 isomer B, suggesting that overexpressed EDEM1 could also trim the C-branch (and likely the A-branch) α1,2Man residue on correctly folded glycoproteins or their folding intermediates. It is important to note that while overexpression of EDEM1 in HEK293 cells resulted in presence of ∼10% (of total *N*-glycans) GlcMan_8_GlcNAc_2_ on NHK during a 3h chasing period following 30 min ^3^[H]Man labeling of HEK293 cells (Hosokawa et al., 2010), overexpression of EDEM3 caused detection of ∼50% (of total *N*-glycans) Man_6–7_GlcNAc_2_ on NHK during a shorter 2 h chasing period (Hirao et al., 2006), revealing that EDEM3 is a much stronger α1,2-mannosidase than EDEM1 in cultured HEK293 cells. Alternatively, the difference in the demannosylation activity of EDEM1 and EDEM3 could be caused by their differential selectivity for certain glycoproteins of cultured cells. The detection of increased amount of Man_6_GlcNAc_2_ *N*-glycans in both EDEM1- and EDEM3-overexpression studies (Hirao et al., 2006) suggested that both EDEMs were capable of demannosylating α1,2-Man residues beyond the C-branch. It is also possible that overexpression of EDEM1/3 could somehow stimulate the activity of the Golgi-localized α1,2-mannosidases responsible for the detected elevation of Man_6_GlcNAc_2_-glycans on total glycoprotein extracts during the ^3^[H]Man pulse-chasing experiments. Thus, it was concluded that the yeast Htm1 is a C-branch-specific α1,2-mannosidase while the mammalian EDEM1/3 preferentially demannosylate the C-branch α1,2Man residue with additional demannosylation activity that removes α1,2Man residues of the A-branch.

## The Arabidopsis MNS4 and MNS5 Are Also Active α1,2-Mannosidase *in vivo*

The Arabidopsis genome encodes two homologs of EDEMs (Mast et al., 2005), which were named MNS4 and MNS5 due to their sequence homology to the yeast Mns1 (Hüttner et al., 2014b) and the presence of three other Mns1-like α1,2-mannosidases known as MNS1, MNS2, and MNS3 (Liebminger et al., 2009). Loss-of-function mutations in MNS4 or MNS5 had no detectable impact on the ERAD of bri1-5 and bri1-9, however, simultaneous elimination of both MNS4 and MNS5 was able to block ERAD of the two ER-retained mutant BR receptors, thus suppressing the dwarf phenotypes of the corresponding Arabidopsis mutants (Hüttner et al., 2014b). The simultaneous elimination of MNS4 and MNS5 also blocked the degradation of an engineered ERAD substrate, SUBEX-C57Y-GFP containing the Cys^57^-Tyr mutated variant (mimicking the *bri1-5* mutation) of the extracellular domain of Arabidopsis STRUBBELIG known to be involved in tissue morphogenesis (Chevalier et al., 2005; Hüttner et al., 2014a). These genetic results provided a strong support for the involvement of MNS4 and MNS5 in a plant ERAD process. Importantly, the suppressive effect of the *mns4 mns5* double mutation on the growth defects of *bri1-5* could be nullified by an *alg3* mutation (Hüttner et al., 2014b), implying that MNS4 and MNS5 function redundantly in generating the conserved *N*-glycan ERAD signal known to mark bri1-5 for ERAD (Hong et al., 2012). The demonstration to show that MNS4 and MNS5 were capable of demannosylating *N*-glycans *in vivo* was carried out in a tobacco transient expression system, which coexpressed MNS4/5-GFP with an ER-resident glycoreporter GCS1-CTS-GFP_glyc_, a GFP-tagged chimeric glycoprotein composed of a short ER membrane-anchored N-terminal fragment of the Arabidopsis GI/GCS1 (GCS1-CTS) and a 217-amino-acid fragment of the human immunoglobulin G1 heavy chain with a single *N*-glycosylation site (Schoberer et al., 2009). It was shown that the single *N*-glycan on GCS1-CTS-GFP_glyc_, when expressed alone, was mainly Man_8–9_GlcNAc_2_. However, coexpression of the glycoreporter with the wild-type MNS4-GFP but not its catalytically inactive variant resulted in a significant increase in Man_7_GlcNAc_2_ (Hüttner et al., 2014b). This glycan was subsequently analyzed by liquid chromatography-electrospray ionization-mass spectrometry with three Man_7_GlcNAc_2_ standards, revealing its identity as the Man_7_GlcNAc_2_ isomer A lacking the B/C-branch α1,2-Man residues. Together, these results clearly demonstrated that MNS4 is a plant C-branch-specific α1.2-mannosidase. Intriguingly, despite functional redundancy in the genetic experiments, the tobacco-expressed MNS5 had no detectable effect on the *N*-glycan of GCS1-CTS-GFP_glyc_. Yet both MNS4 and MSN5 were able to generate the Man_7_GlcNAc_2_ glycan on a soluble glycoreporter GFP_glyc_-HDEL (lacking the GCS1-CTS fragment but carrying the HDEL ER retrieval motif), revealing that both MNS4 and MNS5 are active C-branch-specific α1,2-mannosidases with MNS5 possibly being more selective for its substrates. It is important to note that there has been no report on whether the two tested glycoreporters are correctly folded glycoproteins or are misfolded and degraded via a typical plant ERAD process. It remains to be tested whether MNS4 and MNS5 are folding-sensitive mannosidases that preferentially trim the C-branch α1,2Man residue of misfolded glycoproteins over their native conformers.

## The Mammalian EDEM2 Is a B-Branch-Specific α1,2-Mannosidase *in vivo*

The mammalian EDEM2 is a unique member of the Htm1/EDEM family. An earlier study (Mast et al., 2005) showed that EDEM2 lacked *in vitro* (using purified EDEM2 protein with fluorescent-labeled Man_5–9_GlcNAc_2_ glycans) and *in vivo* mannosidase activity using the same assay (analyzing ^3^[H]Man-labeled *N*-glycan profiles from HEK293 cells transfected with or without EDEM) that demonstrated the *in vivo* mannosidase activity of EDEM1/3. Although two initial studies showed that EDEM2 overexpression stimulated degradation of glycosylated ERAD clients but not their non-glycosylated variants (Mast et al., 2005; Olivari et al., 2005), a later study, which investigated the role of all three EDEMs via siRNA strategy, found that EDEM2 was required for degrading both glycosylated and non-glycosylated variants of the human sonic hedgehog (Tang et al., 2014), which was known to be self-cleaved in the ER with its cleaved N-terminal fragment secreted for signaling and its C-terminal fragment to be degraded through ERAD (Chen et al., 2011). The discrepancy between these two studies could be attributed to different ERAD substrates or experimental approaches, overexpression vs. RNAi-mediated gene silencing that could cause off-target or compensatory effects.

A seminal study that utilized gene knockout instead of siRNA-mediated gene silencing made a surprising discovery of EDEM2 being a major α1,2-mannosidase responsible for the B-branch α1,2Man-trimming reaction in certain cultured cells (Ninagawa et al., 2014). This study was performed with mammalian cell lines different than those used in previously published ERAD studies: DT40 derived from chicken lymphocytes and HCT116 derived from human colonic carcinoma, which were used due to their easier manipulation for creating gene knockouts. Total *N*-glycan analyses of DT40 cells lacking individual members of the ERManI/EDEMs confirmed the previous findings of higher *in vivo* mannosidase activity of EDEM3 than EDEM1 (Ninagawa et al., 2014). Surprisingly, while knocking out ERManI resulted in a slight increase of Man_9_GlcNAc_2_, elimination of EDEM2 elevated the relative abundance of Man_9_GlcNAc_2_ to that of Kif-treated DT40 cells, indicating that EDEM2 is a much stronger B-branch-specific α1,2-mannosidase than ERManI. A low ERManI activity seemed to be consistent with its rapid turnover in cultured mammalian cells (Wu et al., 2007; Termine et al., 2009) and earlier *N*-glycan analyses of cell cultures, which revealed the predominant presence of Man_9_GlcNAc_2_ after 30-min ^3^[H]Man-pulse labeling (Mast et al., 2005; Hirao et al., 2006). In comparison, the major *N*-glycan after 20-min ^3^[H]Man-pulse labeling in yeast cells was Man_8_GlcNAc_2_ (Clerc et al., 2009). However, careful reading of the reported *N*-glycan results revealed increased abundance of several high Man-type *N*-glycans with an intact B-branch, including Man8A (lacking the A-branch terminal α1,2-Man residue), Man7B (lacking terminal α1,2-Man residues of the A and C-branches), and Man6’ (missing all three A/C-branch α1,2-Mman residues) in *ERManI-KO* but not *EDEM2-KO* DT40 cells, confirming the importance of ERManI in *N*-glycan maturation of mammalian cells. This result was similar to what was reported for the total *N*-glycans extracted from an Arabidopsis *mns3* mutant lacking the plant Mns1/ERManI homolog, which accumulated unusual *N*-glycans with an intact B-branch, thus revealing the existence of an alternative pathway for the formation of complex-type *N*-glycans in plants (Liebminger et al., 2009). It is quite possible that the dramatic impact of *EDEM2-KO* instead of *ERManI-KO* on the accumulation of Man_9_GlcNAc_2_ is caused by ER retention of a large number of secretory/transmembrane proteins (due to their inefficient folding/assembly) in cultured mammalian cells (Schubert et al., 2000) and the non-ER localization of ERManI (Benyair et al., 2015a). Thus, EDEM2 is an active B-branch-specific α1,2-mannosidase *in vivo* that likely demannosylate *N*-glycans of ER-retained glycoproteins.

Consistent with the total *N*-glycan data, analysis of ATF6, a known endogenous glycosylated ERAD substrate (Horimoto et al., 2013), revealed that eliminating EDEM2 but not ERManI not only inhibited the degradation of ATF6 but also reduced its SDS-PAGE electromobility similar to that of Kif-treated DT40 cells. By comparison, the electromobility of ATF6 extracted from *EDEM1/3-KO* cells was between that of ATF6 extracted from the mock-treated and Kif-treated DT40 cells (Ninagawa et al., 2014). These are very important results indicating that generating the conserved *N*-glycan signal for a mammalian ERAD pathway (at least in certain cultured mammalian cells such as DT40) also requires the B-branch Man-trimming reaction. The impacts of knocking out ERManI/EDEMs on *N*-glycan profiles and the degradation rates and electromobility shifts of ATF6 were subsequently confirmed in the human HCT116 cell line (Ninagawa et al., 2014). More importantly, the inhibitory effect of *EDEM2-KO* on ERAD and Man-trimming of ATF6 could be rescued by the wild-type EDEM2 but not its catalytically dead mutant. These results demonstrated that EDEM2 is a B-branch-specific α1,2-mannosidase that initiates the demannosylation process for the mammalian ERAD pathway. The resulting Man8B is further demannosylated at its C-branch by EDEM3 and/or EDEM1 to form Man7C with an exposed α1,6Man residue, which can then be recognized by the ERAD lectin OS-9/XTP3-B.

## Demonstration of the α1,2-Mannosidase Activity of Htm1 *in vitro*

The revelation of *in vivo* α1,2-mannosidases activity of the Htm1/EDEMs prompted another round of experiments to demonstrate that the Htm1/EDEMs could demannosylate *N*-glycans *in vitro*, an ultimate test to show that Htm1/EDEMs are *bona fide* α1,2-mannosidases rather than accessary factors of unknown mannosidase. Two hypotheses were put forward to explain the early failure of *in vitro* α1,2-mannosidase assays: Htm1/EDEMs are only active toward *N*-glycans of misfolded proteins and Htm1/EDEMs require one or more cofactors for their α1,2-mannosidase activities.

The first successful demonstration of an *in vitro* α1,2-mannosidase activity was performed with the yeast Htm1 coexpressed with the yeast Pdi1 (protein disulfide reductase 1) in insect cells (Gauss et al., 2011). Pdi1 is the only essential member of the yeast 5-member PDI family and consists of 4 TRXL domains known as a, b, b′, and a′ (a/a′ carrying redox-active motif and b/b′ being redox inactive) (Farquhar et al., 1991; Norgaard et al., 2001). These four TRXL domains form a twisted U-shaped structure with the a/a′ domains forming the two arms, the b/b′ domains establishing the curved base, and an inner hydrophobic surface thought to interact with misfolded proteins (Tian et al., 2006). Pdi1 was shown to covalently interact (via mixed disulfide bridges) with Htm1 in yeast cells (Clerc et al., 2009), required for the generation of a disulfide bond in Htm1 (Sakoh-Nakatogawa et al., 2009), and necessary to produce a soluble Htm1 protein in the insect cells (Gauss et al., 2011). To demonstrate the *in vitro* α1,2-mannosidase activity of a purified Htm1-Pdi1 complex, Gauss et al. used the total protein extracts of ^3^[H]Man-pulse labeled yeast cells of defined genotypes (for producing *N*-glycans of defined structures) as the assay substrates, which were then treated with PNGase F to release *N*-glycans for analysis by high performance liquid chromatography (HPLC). Incubation of the purified Htm1-Pdi1 complex with the protein extracts of the wild-type yeast cells resulted in ∼10% conversion of Man_8_GlcNAc_2_ to Man_7_GlcNAc_2_ (Gauss et al., 2011). Importantly, it was found that reduction/alkylation of the protein extracts was a necessary step to achieve the maximum activity, supporting the hypothesis that Htm1/EDEMs preferentially demannosylate *N*-glycans of misfolded proteins. Consistent with the *in vivo* result showing that Htm1 was only active with the Man8B *N*-glycan (Clerc et al., 2009), the Htm1-Pdi1 complex failed to trim Man_9_GlcNAc_2_ on total proteins extracted from Δ*mns1* yeast cells but was able to convert Man_9_GlcNAc_2_ to Man_7_GlcNAc_2_ when co-incubated with a recombinant Mns1 (Gauss et al., 2011; Liu et al., 2016). This successful *in vitro* assay indicated that Htm1 not only requires a cofactor (Pdi1) but also preferentially demannosylates Man8B of misfolded glycoproteins. It is important to note that the purified Htm1-Pdi1 complex was able to demannosylate *N*-glycans of correctly folded glycoproteins (albeit with reduced efficiency), which was later confirmed by an *in vivo* experiment showing that overexpression of the wild-type Htm1 but not its catalytically inactive mutants resulted in elevated abundance of Man_7_GlcNAc_2_ with a concomitant reduction of Man_8_GlcNAc_2_ on the endogenous Htm1 and other yeast glycoproteins (Pfeiffer et al., 2016).

Because the yeast protein extracts used as the *in vitro* substrates could contain other necessary cofactor(s) required for the α1,2-mannosidase activity of Htm1, two additional *in vitro* studies were performed using *N*-glycans of well-studied single glycoproteins, which could be manipulated to alter their folding status (Liu et al., 2016; Pfeiffer et al., 2016). Consistent with the earlier reports (Clerc et al., 2009; Sakoh-Nakatogawa et al., 2009), epitope-tagged Htm1 proteins expressed in yeast cells were copurified with Pdi1, and the resulting Htm1-Pdi1 complexes were assayed for their *in vitro* mannosidase activity using the ER-retained CPY-HDEL and its mutant variant CPY^∗^-HDEL or bovine pancreatic ribonuclease B (RNase B), which was known to have a single but heterogeneous Man_5–8_GlcNAc_2_ *N*-glycan (Fu et al., 1994) and could be chemically or enzymatically treated to alter its conformations (Ritter and Helenius, 2000; Ritter et al., 2005). As expected, the purified Htm1-Pdi1 complex but not its catalytically dead variants preferentially converted Man_8_GlcNAc_2_ into Man_7_GlcNAc_2_ on CPY^∗^-HDEL or chemically denatured/modified CPY-HDEL over the native CPY-HDEL. Contradictory to what was previously found (Gauss et al., 2011), the Htm1-Pdi1 complex purified from yeast cells could directly trim Man_9_GlcNAc_2_ *N*-glycan (on denatured CPY-HDEL purified from the Δ*mns1Δhtm1* yeast cells) to form the Man_8_GlcNAc_2_ isoform C, providing a direct biochemical support for a previously described Mns1-independent Htm1-dependent mechanism to generate an α1,6Man-exposed Man_8_GlcNAc_2_ *N*-glycan (Hosomi et al., 2010; Chantret et al., 2011). The *in vitro* α1,2-mannosidase activity of the Htm1-Pdi1 complex was further confirmed using RNase B as the substrate. It was found that the Htm1-Pdi1 complex preferentially demannosylated *N*-glycans of the chemically denatured or proteolytically cleaved RNase B, and that incubation of the Htm1-Pdi1 complex but not its catalytically dead mutant with denatured RNase B resulted in a marked reduction of Man_8_GlcNAc_2_ glycans with a concomitant increase in Man_7_GlcNAc_2_ glycans (Liu et al., 2016; Pfeiffer et al., 2016). Consistent with the *in vivo* study (Clerc et al., 2009), ^1^H-nuclear magnetic resonance-based analysis of *N*-glycans of denatured RNase B revealed that the Htm1-Pdi1 complex specifically cleaved the α1,2Man-α1,6Man linkage of the C-branch. The most interesting experiment of the two Htm1 studies of 2016 was the *in vitro* assay of the Htm1-Pdi1 complex using the affinity-purified Man_8_GlcNAc_2_-carrying RNase B (Ritter et al., 2005), which was chemically and enzymatically manipulated to form several well-defined structural conformers with varying degree of unfolding/misfolding. This experiment revealed that the Htm1-Pdi1 complex was a folding-sensitive α1,2-mannosidase that prefers nonnative glycoproteins with partially folded structure over globally denatured glycoproteins. Thus, the Htm1-Pdi1 complex, which marks a terminally misfolded glycoprotein for degradation, is very similar to UGGT that uses a flexible C-shaped substrate binding domain composed of 4 TRXL domains to preferentially recognize and reglucosylate unfolded proteins with partially folded structures (Calles-Garcia et al., 2017; Roversi et al., 2017; Satoh et al., 2017) for sending a misfolded glycoprotein back to the CNX/CRT folding cycle. Together, these three yeast studies have demonstrated that the Htm1-Pdi1 complex was an active α1,2-mannosidase in *vitro* that preferentially demannosylate the C-branch α1,2Man residue of *N*-glycans of misfolded (with partially folded structures) glycoprotens over their native or globally unstructured conformers.

The requirement for Pdi1 as the necessary cofactor of Htm1 was supported by a genetic study that identified a missense *pdi1* allele, *pdi1-1*, which carries a Leu^313^-Pro mutation near the center of the b′ domain thought to be involved in the substrate binding with little impact on its oxidoreductase function (Gauss et al., 2011). The *pdi1-1* mutation disrupts the Htm1-Pdi1 association, reduces the Htm1 stability, and inhibits the Htm1’s *in vivo* mannosidase activity. Importantly, *pdi1-1* greatly reduced ERAD of two glycosylated ERAD clients, and its inhibitory impact on ERAD could be rescued by Htm1 overexpression (compensating for the weaker Htm1-pdi1-1 binding) or Δ*alg3* deletion, indicating that the *pdi1-1* mutation specifically affects the Htm1 activity. Further support for the importance of the Htm1-Pdi1 binding for the Htm1’s α1,2-mannosidase activity came from several transgenic experiments expressing mutant Htm1 variants carrying mutations that disrupt the Htm1-Pdi1 interaction. For example, deleting the C-terminal domain known to be essential for the covalent Htm1-PDi1 interaction (Clerc et al., 2009; Sakoh-Nakatogawa et al., 2009) or just deleting the last 4 amino acids inhibited the α1,2-mannosidase activity of Htm1 in converting Man_8_GlcNAc_2_ to Man_7_GlcNAc_2_ (Liu et al., 2016). Similarly, mutating Phe^632^ into Leu in the C-terminal alpha-helix region of Pro^630^-Trp^636^ inhibited the Htm1-Pdi1 interaction, reduced the Htm1’s demannosylation activity, and compromised the Htm1’s ERAD-stimulatory activity (Pfeiffer et al., 2016). Together, these studies demonstrated an absolute requirement of the yeast Pdi1 for the α1,2-mannosdase activity of Htm1.

Why does Htm1 need Pdi1 for its mannosidase activity? An earlier study showed that the Htm1-Pdi1 interaction is required to form the intramolecular disulfide bridge between Cys^65^ and Cys^445^ of Htm1, which is essential for keeping the folded structure of its MLD (Sakoh-Nakatogawa et al., 2009). This study also showed that Htm1 maintains the covalent (via mixed disulfide bridges involving at least two C-terminal Cys residues, Cys^579^ and/or Cys^644^) and non-covalent interaction (likely requiring a folded MLD structure) with Pdi1 even after the Pdi1-catalyzed formation of the Cys^65^–Cys^445^ disulfide bridge, implying an additional ERAD-supporting role. Treatment of the purified Htm1-Pdi1 complex with a thiol-reactive agent had little effect on its *in vitro* α1,2-mannosidase activity (Liu et al., 2016), indicating that the covalently bound Pdi1 is not engaged in a Cys-mediated biochemical process during the *in vitro* mannosidase assay. Consistently, Δ*htm1* mutation was shown to inhibit the ERAD of both CPY^∗^ and its Cys-free variant CPY^∗^ΔCys (Pfeiffer et al., 2016). Given the similarity between the Htm1-Pdi1 complex and UGGT in recognizing unfolded glycoproteins with partially folded structures and the demonstrated chaperone function of Pdi1 (Wang and Tsou, 1993), it is tempting to speculate that the disulfide bridged Pdi1 uses its 4 TRXL domains with a hydrophobic client-binding inner surface to recognize and bind misfolded glycoproteins, leading to conformational changes in the MLD of Htm1 and activation of its α1,2-mannosidase activity. A detailed structural analysis of a purified Htm1-Pdi1 complex could shed light on the biochemical mechanism by which this disulfide-bridged ERAD “folding sensor” recognizes misfolded glycoproteins, demannosylates their *N*-glycans, and forces their entry into the ERAD pathway.

## Demonstration of *in vitro* Mannosidase Activity of the Mammalian EDEMs

Recent studies have shown that PDI binding is also required for the *in vitro* and *in vivo* α1,2-mannosidase activity of the mammalian EDEMs. The first experiment to demonstrate the *in vitro* mannosidase activity of EDEMs was performed with EDEM3, which has a long C-terminal domain exhibiting no sequence homology with the Htm1’s C-terminal domain known to be essential for its disulfide bridge-mediated interaction with the yeast Pdi1. Despite the fact that it’s *in vivo* α1,2-mannosidase activity was discovered in 2006, the first report for its *in vitro* mannosidase activity was published 12 years later when a FLAG-tagged EDEM3 was found to be copurified with ERp46, a member of the mammalian PDI family that contains three redox-active TRXL domains and can rescue the yeast Δ*pdi1* mutation (Knoblach et al., 2003; Kozlov et al., 2010). Importantly, the EDEM3-ERp46 interaction was quite specific as EDEM3 failed to bind P5 and PDI, two other members of the mammalian PDI family; however, it remains an open question if EDEM3 interacts with additional mammalian PDIs, such as ERdj5 and TXNDC11 (thioredoxin domain-containing protein 11 with 5 predicted TRXL domains) which were known to be involved in mammalian ERAD (Ushioda et al., 2008; Timms et al., 2016). The specific EDEM3-ERp46 binding immediately prompted an *in vitro* mannosidase assay using affinity purified EDEM3 that was expressed alone or coexpressed with ERp46 in HEK293 cells. The purified EDEM3 was assayed for its mannosidase activity with affinity-purified TCRα (the α subunit of the T cell receptor complex), a glycoprotein known to be degraded by ERAD when expressed alone in cell cultures (Tiwari and Weissman, 2001). The reaction mixtures were subsequently separated by SDS-PAGE, and the electromobility shift of TCRα was used to measure the *in vitro* mannosidase activity. It was found that the EDEM3-ERp46 complex exhibited a much stronger demannosylation activity than the EDEM3 expressed alone in HEK293 cells. Importantly, the ability to alter the SDS-PAGE mobility of TCRα was inhibited by Kif treatment or by the catalytically inactive D^294^N mutation with D^294^ corresponding to D^463^ essential for the human ERManI activity (Vallee et al., 2000a), indicating that the EDEM3-ERp46 was an active mannosidase *in vitro*. It should be noted that the purified EDEM3 without coexpressed ERp46 was still capable of demannosylating *N*-glycans of TCRα albeit with a greatly reduced rate; however, such a residual mannosidase activity could be contributed to a small amount of EDEM3 complex formed with the endogenous ERp46 of the cultured HEK293 cells. The role of ERp46 in supporting the *in vitro* and *in vivo* α1,2-mannosidase activity and the ERAD-stimulatory function of EDEM3 requires a stable *ERp46-KO* cell line. *N*-glycan analysis of RNase B or known mammalian ERAD clients should be performed to determine if the EDEM3-ERp46 complex preferentially demannosylates *N*-glycans of misfolded glycoproteins and is a C-branch-specific α1,2-mannosidase or is capable of extensive α1,2Man-trimming *in vitro*.

What could be the biochemical function of ERp46 to support the EDEM3’s mannosidase activity? An initial *in vivo* experiment showed that ERp46 regulates the redox state of EDEM3 and covalently interacts with EDEM3 via disulfide linkages between its three redox-active sites (CGHC) and the Cys^83^ and Cys^442^ residues of EDEM3, which are the equivalent of the Cys^65^–Cys^445^ disulfide bridge of Htm1 and likely form a disulfide bridge due to their spatial proximity in a 3D model deduced from the crystal structures of Mns1/ERManI (Yu et al., 2018; Vallee et al., 2000a, b). Altering the redox state of the reaction conditions had little impact on the *in vitro* mannosidase activity of EDEM3 or EDEM3-ERp46 complex despite alteration of the redox state of the purified EDEM3 (from HEK293 cells without coexpressing ERp46). Consistent with the yeast Htm1-Pdi1 studies, purifying the EDEM3-ERp46 complex in the presence or absence of a thiol-reactive agent had no effect on the *in vitro* mannosidase activity, indicating that the *in vitro* demannosylation reaction of the purified EDEM3-ERp46 complex did not require a Cys-mediated biochemical event. Interestingly, mutating the second Cys residue in all three redox-active CGHC sites, which caused formation of stable disulfide bridged EDEM3-ERp46(CGHA) complexes, had no effect at all on the *in vitro* mannosidase activity. By contrast, mutating all 6 redox-active Cys residues completely inhibited the ERp46-EDEM3 binding and the EDEM3’s mannosidase activity. Together, these experiments showed that a disulfide bridged ERp46 was absolutely required for the mannosidase activity of EDEM3. It remains to be investigated to fully understand the biochemical mechanism by which both the wild-type ERp46 and ERp46(CGHA) promote the EDEM3’s mannosidase activity.

A recent study that examined the *in vitro* mannosidase activity of EDEM1 and EDEM2 suggested that EDEM1/2-interacting PDIs might help alter the conformations of their glycoprotein substrates, thus allowing easy access to their linked *N*-glycans (Shenkman et al., 2018). Both EDEM1 and EDEM2 immunoprecipitated from HEK293 cells were assayed for their *in vitro* mannosidase activity towards free *N*-glycans, which were PNGase F-released from a principal egg yolk glycoprotein vitellogenin of the giant freshwater prawn (*Macrobrachium rosenbergii*) (Roth et al., 2010). Interestingly, both EDEM1 and EDEM2 exhibited very low but nevertheless reproducible *in vitro* activity of converting free Glc_0–1_Man_8_-_9_GlcNAc_2_ glycans into shorter Man_5–8_GlcNAc_2_ glycans (Shenkman et al., 2018), indicating that both EDEMs were active mannosidases capable of extensive Man-trimming *in vitro.* Both EDEMs displayed similar weak activity of converting Glc_0–1_Man_9_GlcNAC_2_ to Glc_0–1_Man_8_GlcNAc_2_ on native vitellogenin. This is a very important finding as it confirmed earlier *in vivo* studies (Hosokawa et al., 2003, 2010) showing that EDEM1 could directly demannosylate Glc_0–1_Man_9_GlcNAc_2_ *N*-glycans. Interestingly, coincubation of EDEM1 with ERManI increased production of Man_7_GlcNAc_2_ glycans on vitellogenin; however, no similar additive effect was observed when EDEM1 was co-incubated with EDEM2, contradicting with the major finding of the EDEM knockout study by Ninagawa et al. (2014), showing that EDEM2 exhibited a much stronger activity than ERManI to cleave the B-branch α1,2Man residue *in vivo*. Importantly, coincubation of EDEM1/2 with at least two EDEM1/2-binding PDIs, PDI (Koivu et al., 1987) and TXNDC11 (Timms et al., 2016), but not ERdj5 known to interact with EDEM1 to enhance ERAD (Ushioda et al., 2008), stimulated the *in vitro* mannosidase activity of the two EDEMs only when vitellogenin was used as the assay substrate (Shenkman et al., 2018). Consistent with the yeast Htm1 studies, both EDEM1 and EDEM2 exhibited stronger *in vitro* α1,2-mannosidase activities toward *N*-glycans of chemically denatured vitellogenin than those of native vitellogenin, trimming Glu_0–1_Man_9_GlcNAc_2_ to shorter *N*-glycans. The most surprising result of the study was the revelation that PDI1 or TXNDC11 had little impact on the *in vitro* mannosidase activity of EDEM1/2 when denatured vitellogenin was used as the assay substrate, leading to a speculation that the EDEM1/2-associated PDI/TXNDC11 was mainly used to alter the conformations of their ERAD clients to maximize accessibility of their *N*-glycans to the EDEMs. It is important to note that, given the demonstrated strong binding between Htm1/EDEM3 with a member of the PDI family, the purified EDEM1/2 might contain covalent EDEM1/2-PDI/TXNDC11 complexes responsible for the detected *in vitro* mannosidase activities. Further investigation, including mutagenesis of the redox-active TRXL domains and treatment of the purified EDEM1/2 with a thiol-reactive alkylating agent, is needed to investigate how EDEM1/2 interacts with PDI/TXNDC11 and whether their *in vitro* mannosidase activities require a Cys-mediated biochemical event.

Indeed, a recent study revealed that EDEM2, when expressed in the *EDEM2-KO* HCT116 cells, formed a disulfide-bridged enzyme complex with TXNDC11 via Cys^692^ of TXNDC11, which contains two redox-active and three redox-inactive TRXL domains (Timms et al., 2016), and Cys^558^ near the C-terminal end of EDEM2 (George et al., 2020). Similar to what was discovered for the yeast Htm1 (Sakoh-Nakatogawa et al., 2009), the EDEM2-TXNDC11 interaction might be important to form the Cys^65^–Cys^408^ disulfide bridge (equivalent of the Cys^65^–Cys^445^ disulfide bridge of Htm1) that is essential for the ERAD-promoting activity of EDEM2. Consistent with the Shenkman et al. (2018) study, the purified EDEM2-TXNDC11 complex, but not the mutant EDEM2(C^558^A) that failed to interact with TXNDC11, was able to demannosylate the free Man_9_GlcNAc_2_ to form Man_8_GlcNAc_2_ (George et al., 2020). HPLC-based *N*-glycan analysis confirmed that the resulting Man_8_GlcNAc_2_ was Man8B lacking the B-branch terminal α1,2Man residue, thus unequivocally proving that EDEM2 is a B-branch-specific α1,2-mannosidase and providing an *in vitro* biochemical support for the earlier cell culture-based knockout experiments (Ninagawa et al., 2014). Importantly, eliminating TXNDC11 not only blocked ERAD of glycosylated ERAD substrates but also completely inhibited Man-trimming of their *N*-glycans measured by their mobility changes on SDS-PAGE. This study not only provided a strong genetic support for a crucial role of the EDEM-PDI binding for their *in vivo* α1,2-mannosidase activities but also supplied additional evidence for the requirement of the B-branch Man-trimming to create the α1,6Man-exposed N-glycan ERAD signal on the C-branch (at least in cultured human HCT116 cells). Together, these recent *in vitro* EDEM studies demonstrated that EDEMs were active α1,2-mannosidases *in vitro* and strongly suggested that their catalytic activities require disulfide bridge-mediated complex formation with members of the mammalian PDI family, which likely function in recognizing and binding of misfolded glycoprotein or altering conformations of glycoproteins to maximize accessibility of their *N*-glycans by the EDEMs.

As discussed above, Arabidopsis genome encodes two Htm1/EDEM homologs, namely Arabidopsis MNS4 and MNS5, which were capable of demannosylating a single *N*-glycan on engineered glycoreporters in a transient expression experiment in tobacco plants. However, it remains to be determined if MNS4 and MNS5 also form stable disulfide bridged protein complexes with members of the Arabidopsis PDI family of 14 PDI-like proteins (Selles et al., 2011) and if so, whether any of the MNS4/MNS5-PDI complexes exhibits *in vitro* α1,2-mannosidase activity with free oligosaccharides or *N*-glycans of misfolded or native glycoproteins.

## The Requirement for the Trimmed B-Branch in Creating the ERAD *N*-Glycan Signal

The genetic studies in yeast clearly demonstrated that the Htm1-catalyzed Man-trimming reaction requires Man8B as its substrate (Clerc et al., 2009), explaining why Δ*mns1* mutation could block degradation of several model glycosylated ERAD substrates (Knop et al., 1996). However, an earlier genetic study revealed the existence of a Htm1-dependent but Mns1-independent ERAD process (Hosomi et al., 2010), which was supported by recent biochemical and metabolic studies showing that Htm1 could directly remove the C-branch terminal α1,2Man residue from the Man_9_GlcNAc_2_ glycan (Chantret et al., 2011; Liu et al., 2016). Contrary to the yeast produced Htm1-Pdi1 complex, the Htm1-Pdi1 complex purified from the insect cells was shown to be only active toward Man8 but exhibited no activity at all toward Man_9_GlcNAc_2_ (Gauss et al., 2011). It is possible that an unknown factor, which is only produced in yeast cells but not in insect cells, is needed to allow the Htm1-Pdi1 complex to directly remove the C-branch α1,2Mman residue of Man_9_GlcNAc_2_. Further studies are needed to fully understand the differential requirement for the Mns1-catalyzed preparatory step on the B-branch for the C-branch trimming activity of Htm1.

The situation of the mammalian ERAD is very confusing. There are two major contributing factors. The first one is related to different cell lines used in various mammalian cell culture studies, including HEK293 cell and its derivative lines, CHO and its glycosylation-defective mutant cell lines, 3T3/NIH-3T3 (derived from Swiss albino mouse embryo), Hep2G, DT40, HCT116. Given the extensive genetic, epigenetic, and transcriptomic variability of different cell lines, these different mammalian cell cultures certainly exhibit huge variability in the protein abundance of α1,2-mannosidases, chaperones, different members of the mammalian PDI family, and different redox states of the ER. These variability make it extremely challenging to formulate a universal model to explain the contributions of different members of the mammalian glycoside hydrolase family 47 (including ERManI, EDEMs, and three Golgi-type α1,2-mannosidases) in terminating futile folding cycles of irreparable misfolded glycoproteins and forcing them into the ERAD process for their complete proteolysis. The second one is related to controversy surrounding the subcellular localization of ERManI. Unlike the yeast Mns1 that relies on a Golgi-localized protein, Rer1p, for its steady state ER localization (Massaad et al., 1999), the mammalian ERManI has been suggested to be localized in the ER-derived quality control vesicles (QCVs at the steady state)/ERQC compartment (under ER stress) or the *cis*-Golgi (Avezov et al., 2008; Pan et al., 2011, 2013; Benyair et al., 2015b). Regardless of the actual ERManI subcellular locations, accessing ERManI for its ERAD-promoting function(s) most likely requires vesicle-mediated ER-QCVs/ERQC compartments or ER-Golgi trafficking. It was thought that ER substrates were colocalized in the QCVs/ERQC compartment where the concentration of ERManI is high enough to support its extensive Man-trimming activity similar to what was previously shown *in vitro* when the recombinant ERManI was present at high concentrations (Aikawa et al., 2012, 2014). The competing theory hypothesizes that the *cis*-Golgi-localized ERManI influences proteasome-mediated degradation of ERAD clients via a catalysis-dependent demannosylation mechanism and non-enzymatic processes involving a conserved decapeptide sequence in the luminal stem domain and the cytoplasmic tail (Iannotti et al., 2014; Sun et al., 2020). It was thought both sequence elements contribute to a Golgi-based quality control system that captures and retrieves escaped ERAD clients back to the ER for their degradation, likely through an *N*-glycan and MLD-independent ERManI-client interaction and a direct ERManI binding to a component of the coat protein complex I responsible for the Golgi-to-ER retrograde transport (Pan et al., 2011, 2013; Iannotti et al., 2014; Sun et al., 2020).

A role of ERManI in the mammalian ERAD was originally postulated from pharmacological studies using inhibitors of α1,2-mannosidases (reviewed in Cabral et al., 2001) and the genetic revelation of a crucial role of Mns1 in the yeast ERAD process (Knop et al., 1996). The experimental support for the hypothesis came from two 2003 ERManI studies (Hosokawa et al., 2003; Wu et al., 2003). Hosokawa et al. (2003) showed that overexpression of ERManI increased the Glc_0–1_Man_9_GlcNAc_2_-Glc_0–1_Man_8_GlcNAc_2_ conversion accompanied by increased production of Man_5–7_GlcNAc_2_ during a 2 h-chasing period of 30 min ^3^[H]Man labeling of cultured HEK293 cells, suggesting that overexpressed ERManI was capable of extensive Man-trimming *in vivo*. Importantly, ERManI overexpression stimulated ERAD, which could be further enhanced by coexpression of EDEM1, and the ERManI-induced stimulatory effect of ERAD could be eliminated by Kif treatment, indicating that the ERAD-stimulatory effect of ERManI requires its mannosidase activity. A similar study was performed in the murine hepatoma cell line Hepa1a, showing that overexpression of ERManI stimulated ERAD in a Kif-sensitive manner (Wu et al., 2003). Two loss-of-function studies performed in 2008 showed that RNAi-triggered ERManI silencing in HEK293 cells greatly inhibited ERAD of NHK and PIZ, another misfolded variant (E^342^K) of α1-antitrypsin known to be degraded by ERAD (Wu et al., 2003), whereas ERManI overexpression markedly enhanced their ERAD (Avezov et al., 2008; Termine et al., 2009). Consistently, analysis of Endo H-released ^3^[H]Man-labeled *N*-glycans from the assayed ERAD substrate (after a 4 h-chasing period) revealed a significant increase of Glc_0–1_Man_9_GlcNAc_2_ glycans (∼55% of total *N*-glycans) in HEK293 cells transfected with an *ERManI-RNAi* construct compared to HEK293 cells transfected with a control RNAi plasmid (<10% of total *N*-glycans being Glc_0–1_Man_9_GlcNAc_2_). As expected, the relative amount of Man_8_GlcNAc*2* was markedly reduced by ERManI silencing (from 30% in the control to 17% in ERManI-silenced cells). Additional support for a role of ERManI in Man-trimming came from analysis of electromobility changes of ^35^[S]Met-labeled PIZ on SDS-PAGE during a 7 h-chasing period, revealing that while ERManI overexpression accelerated rates of PIZ mobility change, ERManI silencing almost completely inhibited the PIZ’s mobility change on SDS-PAGE. More importantly, coexpression of PIZ with EDEM1 in HEK293 cells resulted in a faster rate of mobility change and degradation of PIZ, whereas ERManI silencing completely eliminated the EDEM1-induced changes, providing a strong support for a role of ERManI in EDEM1-catalyzed Man-trimming. However, caution is needed to interpret these results as RNAi-based gene silencing has been known to cause many off-target or compensatory effects.

Further support for a role of ERManI in the mammalian ERAD came from a recent CRSIPR/Cas9-created knockout of ERManI (Zhou et al., 2015), showing that eliminating ERManI in 293T cells (a HEK293 derivative cell line) inhibited NHK degradation; however, experiments similar to those performed in the *ERManI*-RNAi studies are needed to determine the impact of the *ERManI*-knockout on the Man-trimming and degradation of model ERAD substrates, especially when the ERManI-knockout cells are transfected with EDEM1-3. A recent study using a quadruple-knockout CHO cell line (lacking the ERManI and three Golgi-localized α1,2-mannosiadses) revealed that the wild-type EDEM1 (expressed as the full-length protein or just its MLD) but not its catalytically mutant variants was able to trim the *N*-glycans of NHK (measured by Kif-sensitive electromobility changes on SDS-PAGE), suggesting that the *in vivo* α1,2-mannosidase activity of EDEM1 does not require the ERManI-mediated B-branch Man-trimming. Given the recently established role of EDEM2 in cleaving the α1,2Man-α1,3Man linkage of the B-branch (Ninagawa et al., 2014; George et al., 2020), it is important to examine the activities of the endogenous or overexpressed EDEM1/EDEM3 in stimulating ERAD and Man-trimming of *N*-glycans on widely used glycosylated ERAD clients in multiple ERManI/EDEM2-knockout mammalian cell lines. These future experiments will tell if the EDEM1/EDEM3’s C-branch Man-trimming activity requires a B-branch-trimmed Glc_0–1_Man_8_GlcNAc_2_ as their preferred substrate or if EDEM1/3 can directly demannosylate Glc_0–1_Man_9_GlcNAc_2_ under certain experimental conditions, in certain cultured mammalian cells, and for certain ERAD substrates. Results from these experiments will certainly enhance our understanding of the biochemical functions of ERManI and the three EDEMs in the mammalian ERAD pathway.

The Arabidopsis has an ortholog of the Mns1/ERManI (known as MNS3) that was previously demonstrated to be a B-branch-specific processing α1,2-mannosidase (Liebminger et al., 2009). Loss-of-function *mns3* mutation was known to interfere with the Golgi-mediated *N*-glycan processing as the *N*-glycans on mature glycoproteins all display an intact α1,2Man-α1,3Man B-branch (Liebminger et al., 2009). Importantly, loss-of-function mutation of MNS3 or simultaneous elimination of MNS3 and two Golgi-localized α1,2-mannosidases (MNS1 and MNS2) did not rescue the dwarf phenotype of *bri1-5* and *bri1-9* (Hüttner et al., 2014a), implying that MNS1-3 are not involved in the ERAD of the two ER-retained mutant BR receptors. Similar to what were discovered for the mammalian ERManI (Pan et al., 2011, 2013), the Arabidopsis MNS3 was found to be retained in the *cis*-Golgi by a signal motif (Leu^5^ProTyrSer) localized in the N-terminal cytoplasmic tail as mutating the hydrophobic Leu residue could relocate MNS3 to the ER (Schoberer et al., 2019). More importantly, relocation of MNS3 from the *cis*-Golgi to the ER could enhance the dwarf phenotypes of the *bri1-5* mutant, presumably due to its mannosidase activity in the ER that enhanced ERAD of bri1-5. However, it remains to be determined if the forced ER accumulation of MNS3 stimulates the B-branch α1,2Man-trimming or directly enhances the C-branch α1,2Man cleavage as an earlier study showed that increasing MNS3 concentration resulted in increased conversion of Man_8–9_GlcNAc_2_ to Man_5–7_GlcNAc_2_ in an *in vitro* mannosidase assay (Liebminger et al., 2009). Thus, the current literature on the Arabidopsis α1,2-mannosidases supports that none of the three Golgi-localized α1,2-mannosidases (MNS1, MNS2, and MNS3) is involved in a well-studied plant ERAD pathway and the MNS4/5-mediated generation of the *N*-glycan ERAD signal unlikely requires the B-branch Man-trimming step.

## Conclusion and Future Challenges

Despite rapid progress in structural understanding of the ERAD machinery itself (Schoebel et al., 2017; Eldeeb et al., 2020; Wu et al., 2020), our knowledge of the initial events that commit an irreparable misfolded glycoprotein to ERAD remains incomplete. It was quite clear from the early days of ERAD research that demannosylation constitutes a key step of the ERAD pathway and intensive/extensive investigation in the last quarter century have identified and biochemically characterized the α1,2-mannosidases, which generate the evolutionarily conserved ERAD *N*-glycan signals with exposed α1,6Man residue, and the ERAD lectins that recognize and bind such a conserved *N*-glycan signal. However, the detailed biochemical mechanism by which eukaryotic cells make the end-of-life decision for an irreparable misfolded glycoprotein remains a mystery. The latest discoveries of the requirement of covalent binding to members of the PDI family for the mannosidase activities of Htm1/EDEMs and the structural preference of the yeast Htm1-Pdi1 complex for compact but partially unstructured glycoproteins over globally unstructured conformers suggested a mechanistic mimicry between the Htm1/EDEM-PDI complexes and UGGT, which likely compete for their binding to a misfolded protein, resulting in *N*-glycan demannosylation for degradation and *N*-glycan reglucosylation for another refolding attempt, respectively. Structural studies of a covalently bridged Htm1/EDEM-PDI complex coupled with biochemical and genetic experiments are needed to test this hypothesis and could determine if and how the PDI-mediated substrate binding is structurally coupled with the catalytic activity of the disulfide-bridged Htm1/EDEMs. The past research progress of plant ERAD studies have demonstrated that the two Htm1/EDEM homologs of the Arabidopsis function redundantly in generating the conserved *N*-glycan ERAD signal; however, it remains to be demonstrated if MNS4/5 require covalently bound PDIs for their mannosidase activity *in vitro* and *in vivo*. Proteomics studies, reverse genetics, and *in vitro* biochemical experiments will greatly expand our knowledge of the biochemical mechanism by which the plant Htm1/EDEM homologs recognize misfolded glycoproteins to demannosylate their *N*-glycans, thus tagging them for their elimination via the ERAD pathway.

## Author Contributions

JZ, JW, LL, and JL discussed the writing plan. JZ and JW drafted the manuscript. LL and JL edited the manuscript. All the authors contributed to the article and approved the submitted version.

## Conflict of Interest

The authors declare that the research was conducted in the absence of any commercial or financial relationships that could be construed as a potential conflict of interest.
